# Technologies for circulating tumor cell separation from whole blood

**DOI:** 10.1186/s13045-019-0735-4

**Published:** 2019-05-14

**Authors:** Petra Bankó, Sun Young Lee, Viola Nagygyörgy, Miklós Zrínyi, Chang Hoon Chae, Dong Hyu Cho, András Telekes

**Affiliations:** 1 Department of Biochemical Engineering, Budapest University of Technology and Economics, Budapest, Hungary; 2 Department of Radiation Oncology, Chonbuk National University Hospital, Jeonju, Republic of Korea; 3 Research Institute of Clinical Medicine of Chonbuk National University-Biomedical, Research Institute of Chonbuk National University Hospital, Jeonju, Republic of Korea; 4 Celldi Kft, Budapest, Hungary; 5 Laboratory of Nanochemistry, Department of Biophysics and Radiation Biology, Semmelweis University, Budapest, Hungary; 6 Department of Obstetrics and Gynecology, Chonbuk National University Hospital, Jeonju, Republic of Korea; 7 Department of Oncology, St. Lazarus Hospital, Salgótarján, Hungary

**Keywords:** Cancer, CTC, Circulating, Tumor cell, Whole blood, Separation

## Abstract

The importance of early cancer diagnosis and improved cancer therapy has been clear for years and has initiated worldwide research towards new possibilities in the care strategy of patients with cancer using technological innovations. One of the key research fields involves the separation and detection of circulating tumor cells (CTC) because of their suggested important role in early cancer diagnosis and prognosis, namely, providing easy access by a liquid biopsy from blood to identify metastatic cells before clinically detectable metastasis occurs and to study the molecular and genetic profile of these metastatic cells. Provided the opportunity to further progress the development of technology for treating cancer, several CTC technologies have been proposed in recent years by various research groups and companies. Despite their potential role in cancer healthcare, CTC methods are currently mainly used for research purposes, and only a few methods have been accepted for clinical application because of the difficulties caused by CTC heterogeneity, CTC separation from the blood, and a lack of thorough clinical validation. Therefore, the standardization and clinical application of various developed CTC technologies remain important subsequent necessary steps. Because of their suggested future clinical benefits, we focus on describing technologies using whole blood samples without any pretreatment and discuss their advantages, use, and significance. Technologies using whole blood samples utilize size-based, immunoaffinity-based, and density-based methods or combinations of these methods as well as positive and negative enrichment during separation. Although current CTC technologies have not been truly implemented yet, they possess high potential as future clinical diagnostic techniques for the individualized therapy of patients with cancer. Thus, a detailed discussion of the clinical suitability of these new advanced technologies could help prepare clinicians for the future and can be a foundation for technologies that would be used to eliminate CTCs in vivo.

## Background

Currently, cancer-related illnesses are one of the leading health issues worldwide, causing approximately eight million deaths each year [1], and it is predicted that this number will rapidly increase in the future [2]. Therefore, research on cancer treatment and early diagnostic techniques is vitally important. Innovative modern technologies are capable of performing previously unachievable tasks. Due to these developments, the main diagnostic and treatment methods for patients with cancer are gradually shifting from conventional standards to personalized techniques [3–6]. Moreover, current diagnostic technology has led to the rapid development of precision medicine and molecular diagnostics [7, 8]. Regulated cell division is a physiological process that occurs in all tissues under many different circumstances and microenvironments. Under normal conditions, both cell division and cell death processes are maintained by strictly controlled systems to ensure the integrity of each organ and tissue through a balance between proliferation and programmed apoptosis. However, uncontrolled cell division can lead to the development of malignant tumors. During the process of carcinogenesis, normal cells are typically transformed into cancer cells in a step-by-step manner, and this process interferes not only with normal cell proliferation but also with the normal state of the cell.

While most DNA mutations are not associated with cancer, certain DNA mutations can lead to the development of cancer. DNA mutations are one of the causative factors in the mechanism of cancer development [9, 10]. The biochemical characteristics of the surface of malignant cancer cells are different from those of normal cells. Based on various changes, such as the generation of new surface antigens, proteoglycans, glycolipids, and mucus, modified cancer cells can be distinguished from normal cells. At the cellular level, these alterations in cancer cells compared to normal cells are mainly due to genetic modification and gene expression [11, 12]. When normal cells become cancer cells, they extensively change their microenvironments. For example, cancer cells can initiate angiogenesis to obtain a sufficient blood supply for cell growth and metastasis [13, 14]. However, most vascular circulating cancer cells are removed by immune cells, NK cells, and vascular endothelium or suffer impacts in small vessels; thus, only a small fraction of circulating tumor cells (CTCs) can cause metastasis [15–18].

Obtaining cancer cells that are in circulation is very important for the early diagnosis and prognosis of patients with cancer. In fact, CTCs are separated from primary tumor cells and circulate throughout the body in the vascular system. CTCs play an important role in metastasis formation [19–22]. It is also expected that the detection and analysis of CTCs obtained from the blood could contribute to the diagnosis and treatment of cancer. Recently, a noninvasive diagnostic method, which is named a liquid biopsy, of CTCs has emerged as a very promising new technique for early cancer diagnosis [23, 24]. Clinically, compared to conventional biopsies, this method is an innovative and significantly less invasive technique for obtaining tumor cell samples, and it is expected to be well suited for early cancer diagnosis and for therapeutic decision making [25]. Liquid biopsy techniques can provide real-time information regarding patient staging (metastatic vs. nonmetastatic) and the molecular profile of the tumor. Moreover, liquid biopsies can be repeated with the desired frequency for close monitoring of progress and treatment [26]. Currently, CTC technology is being commercialized on several technological platforms. However, the results of early CTC technical equipment are not standardized; therefore, the obtained data may vary using different equipment. Standardization seems quite difficult due to different devices using various techniques for CTC detection, which causes physicians to be uncertain of the value of this new technology. In the future, standardization of CTC-based technologies will be necessary when increasing amounts of CTC-based products are commonly used in the clinic [27, 28].

In this review, we focus on CTC technologies using whole blood samples without pretreatment since we predict that this approach will be used more commonly for clinical testing in the future. The methodological advantages of this approach make it suitable for clinical use. The applications and significance of CTC technologies in cancer detection and treatment monitoring are discussed along with their documented performance in recent immunoaffinity, size-based, and combined methods (developed to date) with possible future research studies. We also provide detailed information on the latest CTC-based technologies along with their clinical use. This article may be a helpful guide for cancer research scientists and oncologists for distinguishing between the many CTC separation techniques. A better understanding of CTC-based technologies and expected future developments will hopefully improve the treatment and diagnosis of patients with cancer.

## The importance of CTC separation technologies using whole blood samples

In recent years, several techniques have been used and applied for CTC detection, enrichment, and counting. These techniques are based on different methods and target distinctive physical (size, density, etc.) or biological (tumor markers) characteristics of extremely rare CTCs that are found in the blood of patients with cancer.

Considering how different methods have evolved, there are several approaches that could serve as a basis for the grouping and evaluation of existing devices, such as the distinguishing method of the cells, enrichment mode, sample size, purity, cell viability, and sample treatment.

The common description differentiates between immunoaffinity-based and size-based methods, with several subgroups, such as immunomagnetic or microfluidic, for each group. New devices using these techniques are constantly being developed with increased performance.

Since the only Food and Drug Administration (FDA)-approved technique with prognostic value to date is immunoaffinity-based CellSearch, it is important to highlight the advantages and disadvantages of other methods, compare these methods to each other, and compare these methods to the “etalon” CellSearch method even if these methods use different CTC separation techniques. To compare different methods and describe device performance, it is accepted to use the following parameters: capture efficiency, enrichment, purity, throughput, cell viability, and release efficiency [29]. Capture efficiency describes the efficiency by which a device captures CTCs from a sample. Enrichment is similar to the capture efficiency but refers to an increase in tumor cells in the sample volume. Purity indicates the capability of a device to specifically capture CTCs within a background of interfering cells. Throughput refers to the volume or number of cells from a sample that a device can process in a unit of time, and cell viability indicates the number of CTCs that remain alive after capture. Release efficiency describes the number of cells that are recovered [30].

Due to the possibility that CTC determination will become a routine clinical test in the future, quick and easy sample handling and measurements are important. To introduce CTC counting for widespread clinical use, it is advantageous to use CTC devices that require less complicated steps, more automatization, and less-trained laboratory staff. Considering these factors, we found that methods that eliminate the pretreatment step of the clinical samples have a significant effect due to less time consumption and the absence of multiple pretreatment steps. Using untreated whole blood samples is useful for future applications. It has also been reported that centrifugation, lysis, or other pretreatments before a measurement may significantly decrease the detectable number of CTCs in blood samples. Since every milliliter of blood contains more than 10^9^ red blood cells compared to a few CTCs, it is especially important to capture most of the CTCs without interference [30].

Using whole blood presents some challenges for size-based microfluidic methods, especially microfluidic devices, due to membrane clogging by a high concentration of blood cells. To overcome this problem, devices with various pore sizes and shapes have been designed [31–33], or other solutions, such as a fluid-assisted separation technology from Clinomics [34] or the CTC-iChip that combines three technologies to separate CTCs from whole blood by different parameters [35], have been developed.

Methods using immunoaffinity-based (or immunomagnetic) separation typically discriminate CTCs by targeting surface antigens or removing background cells by targeting antigens that CTCs lack. However, the heterogeneity of antigens that are present on the surface of CTCs is part of the challenge associated with separation, and to date, no universal CTC antigens have been identified [30]. Moreover, according to recent studies, the diversity of CTCs is greater than previously presumed, which makes it challenging to capture representative populations and requires further improvement of currently used CTC devices and separation methods [36–39]. Although the use of positive enrichment immunoaffinity-based methods typically results in high purity, these methods usually require the centrifugation of blood samples or red blood cell lysis, which can cause the loss of CTCs [30].

For the validation of several CTC isolation techniques, cancer cell line-spiked blood or buffer samples are frequently used for research purposes, which could be a source of error for both approaches. However, the sizes and antigen expression of these cells differ from those of CTCs; therefore, this method can be used only for preliminary validation and examination of a specific method before using blood samples (containing CTCs) that are obtained from patients with cancer for clinical validation and further application [40]. Therefore, clinical application of CTC devices requires validation of the different techniques and possible standardization using blood samples from patients with cancer (liquid biopsies) after validation with cancer cell line-spiked blood samples [41].

CTC techniques could still be improved, but new and better techniques are constantly being developed. In addition to treatment monitoring and prognosis prediction, it is expected that CTCs will have an important role in the early detection of cancer. Recent studies have already provided some promising examples of the use of CTCs for this purpose [42]. These findings can be extremely helpful for the early diagnosis of some cancer types when a cancer is asymptomatic or there is no available routine screening method. Clinical studies have also shown that CTC counting can provide relevant prognostic information to patients and physicians [30]. Several studies have shown that CTC counting combined with a detailed genetic analysis using cell-free DNA (cfDNA) profiling could provide a more specific description of the progress and prognosis of patients with cancer and provide new information, such as sensitivity or resistance to certain chemo- or biological therapies, to further improve treatments [43–46]. However, not all CTC techniques are suitable for subsequent downstream methods, such as DNA analysis. Therefore, it is important to list the methods that result in label-free viable cells with high purity and better recovery rates (Tables 1 and 2).

**Table 1:** A comparison of all the currently available CTC methods using whole blood, where each method is compared with the other methods and with the only FDA-approved technique. From a clinical perspective, we found the data describing the clinical detection to be the most important. This table shows the immunoaffinity

Separation category subcategory	Technology	Company	Selection criteria	Key features	Capture efficiency	Purity	Recovery	Viability	Sample volume	Throughput	Clinical detection
Immunoaffinity	CellSearch^a^ [47–49]	Menarini-Silicon Biosystems	EpCAM	FDA approved for advanced breast, prostate, and colorectal cancers; ferrofluid nanoparticles; cannot process whole blood			≥ 85%	Nonviable	7.5 ml		71.4% (35/49)
Immunomagnetic positive enrichment	MagSweeper [50]	Stanford University	EpCAM	High-purity live cells	62–70%	> 50%		Viable	9 ml	9 ml/h	100% (17/17)
	MACS [51]	Miltenyi Biotech	EpCAM	Pos/neg enrichment; high surface area to volume; difficult to use with whole blood				Viable			
	IMS [52]		EpCAM	Low background leukocytes (not tested on clinical samples)	84–100%			Mostly viable			
	Strep-tag [53]	Wuhan University	EpCAM, HER2, EGFR	Uses antibodies simultaneously; low sample volume	79%	N/A	70%	85%	1 ml	N/A	100% (17/17)
Microfluidic positive immunocapture	HTMSU [54–56]		EpCAM	Single-step separation; low volumes of blood; conductivity-based enumeration		100%	96%	80%	1 ml	1–2 ml/h	No clinical trial
	CTC-Chip [57]		EpCAM	Micro vortex increases the efficiency; various CTC-specific antigens can be used	65%	52–67%	> 60%	Viable	2.7 ml (average)	1–2 ml/h	99% (115/116)
	GEDI chip [58]		PSMA/HER2 (+ size selection)	Functional assays in situ; size and collision inclination dependency	85%	68%		Viable	1 ml		
Microfluidic immunocapture + nanomaterials	GO chip [59–61]		EpCAM	Graphene oxide nanosheets; easy fabrication; high purity	84–95%	High	91–95%	92%	1 ml	1–3 ml/h	67–100% (2/3, 8/10, and 20/20)
	Microfluidic SiNP platform [62]		EpCAM	Antibody-coated silicone nanopillars for capture enhancement; 1.5–3.0-psi pressure			> 95%		1 ml	1 ml/h	77% (20/26)
	NP-HBCTC-Chip [63]		EpCAM, HER2, EGFR, or cocktail	Antibody-coated gold nanoparticles for capture enhancement	> 90%		91–92%	87–93%	3.5 ml	1 ml/h	100% (4/4)
Negative immunomagnetic enrichment	EasySep [64]	StemCell	CD45	Easy-to-use batch separation; high background	79%	42%			0.5–2 ml	1–4 ml/h	No clinical trial

^a^Included for reference purpose

**Table 2:** A comparison of all the currently available CTC methods using whole blood, where each method is compared with the other methods. From a clinical perspective, we found the data describing the clinical detection to be the most important. This table shows size-based separation, density-based separation, and combined separation methods

Separation category subcategory	Technology	Company	Selection criteria	Key features	Capture efficiency	Purity	Recovery	Viability	Sample volume	Throughput	Clinical detection
Size-based separation								Viable			
Membrane microfilters	FMSA [32, 65–67]		8-μm pores	Extraction with reverse flow; easy modification for application; 1-in. WC pressure	90%	74%	90%	Viable	7.5 ml	45 ml/h	76% (16/21)
	Crescent-shaped trap [68, 69]		Two 5-μm gaps	Simple operation; transparent membrane; real time changes to the flow characteristics; 5–15-kPa pressure	80%	83%	95–96%	N/A	2 ml	0.7 ml/h	100% (5/5)
	SB [33]		8-μm holes on bottom, 40-μm holes on top	Capture is achieved by a gap between the top and bottom porous membranes; reduction in mechanical stress	78–83%			71–74%	1–7.5 ml		
	FAST [34]	Clinomics	8-μm pores + stably-held liquid	Simple use; ultrafast cell enrichment; transparent membrane; 1-kPa pressure	96%	> 2.5 log depletion	96%	Viable	3 ml	180 ml/h (3 ml/min)	83.3% (15/18)^a^
Microfluidic sorting	Parsortix [70, 71]	Angle	10–4.5-μm gap size	Easy operation; multiple use; 99-mbar pressure	42–70%		54–69%	99%	4 ml	10 ml/h	38.5% (10/26)
	MCA [72, 73]		8-μm circular cavities; or 5–9 × 30-μm or 8 × 100-μm rectangular cavities	Integrated enumeration, staining, and washing	80–97%		70–96%	98%	1–3 ml	12 ml/h	80% (40/50)
Density-based separation	OncoQuick [74]	Greiner Bio-One	Density	Elimination of lymphocytes and mononuclear cells; separation media for additional separation			87%	N/A			23% (14/61)
	AccuCyte [75]	RareCyte	Density	Sequential density fractionation; automated			90%	N/A			81% (22/27)
Combined methods	CTC-iChip [76]		EpCAM/CD45 + size	Two operation modes; long setup time; can be automated; 140-kPa pressure	77–98%	Positive, > 3.5 log depletion; negative, > 2.5 log depletion	99.50%		10 ml	8 ml/h	Positive mode, 90% (37/41)
	Negative-negative microfluidic platform [77]		CD45 + size	Negative immunomagnetic enrichment and negative selective microfluidic chip without sample transfer		90%	90%		2 ml	2 ml/h	100% (15/15)

^a^Nonmetastatic patients

The abovementioned CTC methods are expected to radically change cancer detection and treatment by early diagnosis, metastasis detection, and precision medicine methods. Among currently existing methods, we have chosen to describe devices that use whole blood samples and can thus be implemented more easily in clinical practice.

## Technologies for CTC enrichment from whole blood samples

**Fig. 1:**
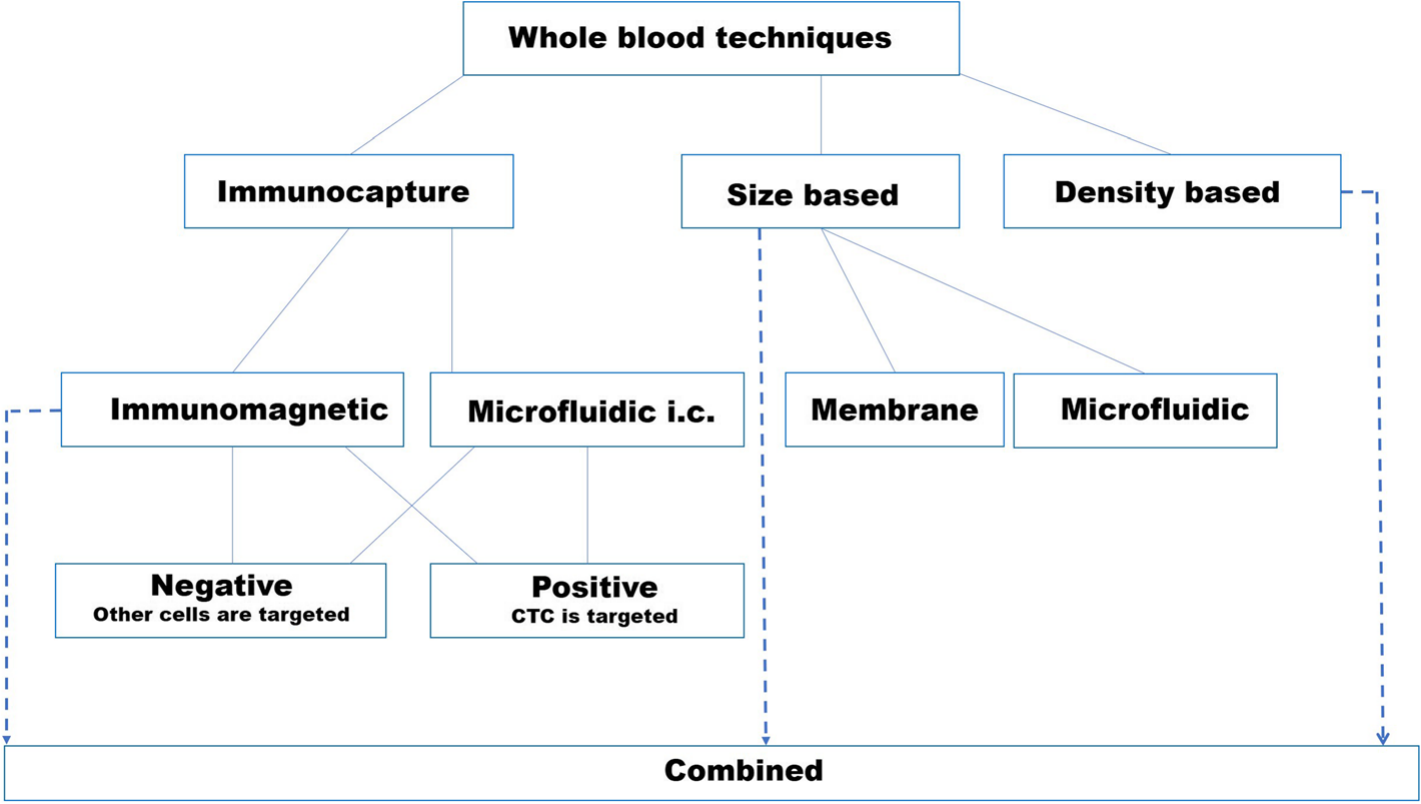
Different types of whole blood methods where immunocapture and physical selection (size and density) methods are separated into subgroups depending on the main properties of the technique. (“Immunoaffinity,” “Size based,” “Density based,” “Combined” sections; i.c immunocapture)

**Fig. 2:**
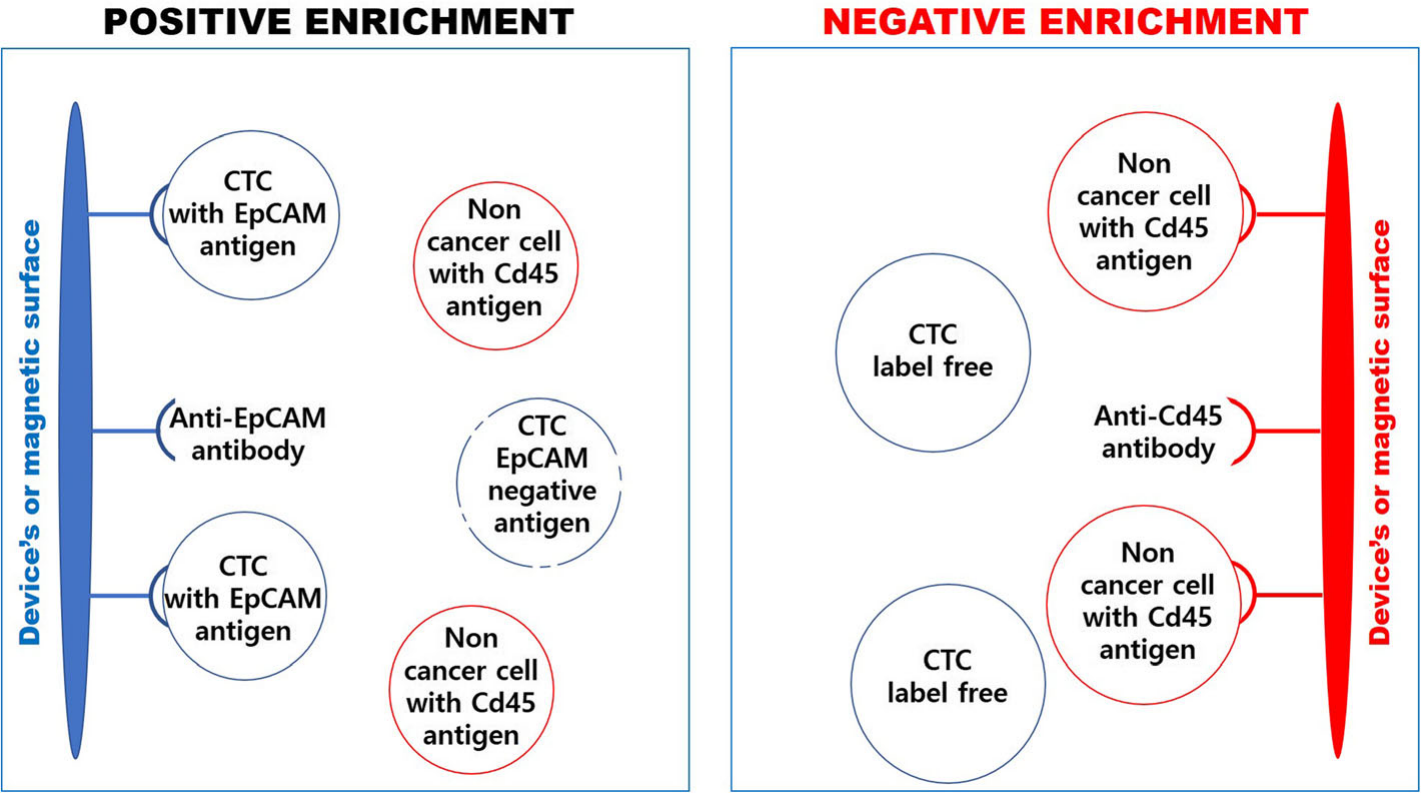
Immunoaffinity-based techniques where the EpCAM antigen is typically targeted on the surface of CTCs for positive enrichment, while the CD45 antigen is targeted on the surface of noncancerous cells for negative enrichment. For positive enrichment, only a subpopulation with a distinct antigen is captured. For negative enrichment, the obtained cancer cells are label-free, and a heterogeneous CTC population is obtained

To support an easier comparison of the following methods, we showed the most important properties of each method (based on availability) in Tables 1 and 2. All the following methods are included in Tables 1 and 2 and are grouped in the same manner as they are grouped in part 3 and in Figs. 1 and 2.

### Immunoaffinity

Immunoaffinity-based CTC techniques are among the first methods developed for capturing CTCs [78], and these methods use specific antigens that are expressed on the surface of CTCs that are not expressed on other cells. For the separation of CTCs from other blood cells, specific antibodies are used to target surface antigens. When tumor cells are captured, the technique is classified as positive enrichment; however, negative enrichment, where antigens that are not expressed on CTCs but are expressed on other blood cells are marked, can also be used, as shown in Fig. 2. Antibodies that are used to capture tumor cells are typically bound to a surface in the device, resulting in difficulties in retrieving captured cells after enrichment or recovering cells from magnetic particles (immunomagnetic method). For negative enrichment, the CD45 antigen is generally targeted to capture normal cells that are found in the blood proximal to CTCs. Negative enrichment methods typically result in lower purity than positive enrichment methods; however, the advantage of negative enrichment methods is that label-free CTCs can be obtained independent of their specific antigen expression [79–81]. Generally, for positive enrichment methods, one type of antigen, the epithelial surface tumor marker (EpCAM), is targeted and typically results in a high-purity separation depending on the antigen. With this method, only CTCs originating in epithelial tumor (breast, colon, prostate, and lung) scans are captured [82], but in recent studies, nonmalignant epithelial cells with the same antigen characteristics have been found in patients with benign colon [82], pancreatic [83], and breast [84, 85] diseases. Nonmalignant tumor cells that have similar characteristics as epithelial CTCs may result in false-positive results; thus, it is also important to consider epithelial-to-mesenchymal transition (EMT) and stem cell markers. To overcome this challenge and include more CTC subpopulations during separation, some recent alternative techniques utilizing surface markers such as epidermal growth factor receptor (EGFR), human epidermal growth factor receptor 2 (HER2), and mucin 1 (MUC1) have been developed. Multiple antibodies have also been used to target stem cell markers and mesenchymal markers [86].

Two of the main disadvantages and challenges of immunoaffinity-based CTC isolation methods described are (1) the heterogeneity of the CTCs, which can cause a loss of CTC subpopulations during enrichment and capture, and (2) CTCs bound to the surface of a device can cause difficulties in cell recovery.

#### Immunomagnetic positive enrichment

Immunomagnetic strategies have overcome one of the challenges caused by surface-bound immunoaffinity methods, namely, difficulties in retaining cells after enrichment for downstream analysis. In typical devices, cells are difficult to recover without the use of trypsin, which likely cleaves surface antigens [59, 87]. This problem can be avoided with immunomagnetic methods where CTCs are immobilized on magnetic beads. Thus, we are focusing on innovative immunomagnetic methods that use whole blood samples since recent studies have shown that pretreatments, such as centrifugation or red blood cell lysis, can cause a reduction in the CTC capture efficiency and result in CTC loss [30, 77].

One of these immunomagnetic technologies that uses whole blood is MagSweeper, which can isolate CTCs with relatively high purity without centrifugation or red blood cell lysis [88]. This device captures CTCs from liquid samples using round-bottom neodymium magnetic rods covered with removable ultrathin (25 μm) nonadherent plastic sleeves, which allow for multiple capture and release cycles, enabling high purity isolations and a high capture efficiency of the device [88, 89]. A robotically controlled magnetic rod isolates CTCs by sweeping through wells containing labeled samples [88]. The sheathed rod is robotically driven to sweep through a well containing a sample in a pattern that covers the entire well area to maximize the capture efficiency of magnetically labeled cells. After washing away contaminating unlabeled cells, labeled cells held by the rod-sheath assembly are moved to a release well where an external magnetic field releases the labeled cells [88]. MagSweeper can process blood at a rate of 9 ml/h, and it can be easily scaled up to process multiple samples in parallel using an array of magnetic rods controlled by a single automated system. The device has been tested on cancer cell line-spiked blood samples using the EpCAM antibody, and the capture efficiency was 62 ± 7% with a purity of 51 ± 18% [88]. Additional molecular analysis of cell lines verified that the capture process did not perturb gene expression. The system has been validated using clinical samples where CTCs were isolated from all 17 patients with metastatic breast cancer, and no CTCs were found in the blood of five healthy donors [88]. A similar validation has also been carried out and showed that the system captures only epithelial cancer when the EpCAM antibody is used, and CTCs are not captured from healthy donors or patients with lymphoma, which is a nonepithelial cancer [41]. The high purity levels obtained by MagSweeper make it suitable for downstream genomic analysis, such as genetic profiling of CTCs in breast cancer [41], single cell detection of phosphatidylinositol 3-kinase catalytic (PIK3CA) mutations in CTCs, and breast cancer metastases [50]. MagSweeper is a multipurpose device, and in addition to capturing CTCs, it can also be used to isolate fetal stem cells or immune cells from fluid or tissue suspension samples [89].

Another enrichment technology based on immunomagnetic separation is a magnetic cell separation system (MACS) [51]. MACS uses high-gradient magnetic separation to capture cells labeled with magnetic nanoparticles conjugated to EpCAM antibodies for enrichment. A sample is passed through a column filled with plastic-coated (to avoid damage to cells) ferromagnetic stainless steel wool that can be magnetized and demagnetized with an external magnetic field, ensuring a sensitive filter for magnetically labeled cells [30, 51]. Cells labeled with superparamagnetic beads are magnetic in a magnetic field and bind to the steel wool fibers. When an external magnetic field is eliminated, the steel wool is demagnetized; therefore, the superparamagnetic particles/cells are no longer bound and can be eluted [51]. Although it has been reported that MACS can be used with some difficulty on whole blood samples and is better suited for tissue samples [52], studies have used MACS to capture CTCs from patients with metastatic cancer [90, 91].

To overcome the difficulty of the MACS technique when using blood samples, another technology, biomimetic immuno-magnetosomes (IMSs), has been recently introduced [52]. For this technique, magnetic nanoclusters (MNCs) are used to overcome the problem of providing a sufficiently high magnetic field for separation without increasing the size of magnetic particles or using a high-gradient external magnetic field. For this method, magnetic nanoclusters are camouflaged with leukocyte membrane fragments, resulting in a magnetosome (LMNC) that suppresses nonspecific leukocyte adsorption in a peripheral blood sample due to its homology, thus decreasing the interference of CTCs with contaminating background cells [92]. In the system, the leukocyte membrane is pre-engineered with azide (N3) to interact with a high-activity dibenzocyclooctyne group-modified antibody (DBCO-Ab) at a controllable density with good fluidity. EpCAM is a CTC detection antibody that has been conjugated to the surface of LMNCs. The resulting IMSs showed high epithelial CTC recognition efficiency [52] with hardly any background cells remaining, which is one of the main advantages of this method. With this technique, 70–90% of rare tumor cells can be captured from whole blood in 15 min [52]. Although no clinical sample tests have been performed, this device has been tested on experimental samples. A negligible number of leukocytes has been measured in samples with up to a 10^5^ ml^−1^ concentration of CTCs, which is a much higher concentration of these rare cells than that in actual clinical samples, suggesting that the number of contaminating leukocytes is increased in clinical blood samples [52].

The Strep-tag® integrated immunomagnetic separation system is an immunomagnetic strategy for reversibly capturing and releasing CTCs by biotin-triggered decomposable immunomagnetic beads, where different antibodies can be used simultaneously to capture more CTC subpopulations [53]. In this system, active Strep-tag II-derived immunoglobulin G (IgG) is fabricated by chemical conjugation and can be reversibly loaded to Strep-Tactin-coated magnetic beads (STMBs) and discharged from STMBs by competitive binding between Strep-tag II and d-biotin towards Strep-Tactin. A high capture efficiency (79%) of CTCs can be achieved using IgG-STMBs because IgG-STMBs can immobilize multiple primary antibodies, such as anti-EpCAM, anti-HER2, and anti-EGFR. To release captured cells, a biotin treatment is used, and it can release 70% of captured cells with high viability (85%). Using this application, CTCs have been isolated from 100% (17 patients) of peripheral blood samples from patients with cancer with high purity, and rare CTCs in whole blood samples can be detected and identified by routine immunostaining. This strategy is recommended for CTC enumeration and molecular profiling of the released viable cells for cancer diagnosis and therapy [53].

#### Microfluidic immunocapture positive enrichment

Microfluidic devices, which are created by microfabrication methods, contain structures that are comparable to the cell length scale. These devices allow for the precise control over sample flow, which is important since this affects cell-antibody contact and therefore the cell capture efficiency [30].

A microchip-based high-throughput micro sampling unit (HTMSU) is a microfluidic device that separates CTCs from the blood using surface-immobilized monoclonal antibodies to target unique membrane proteins. CTCs are retained on monoclonal antibody-coated walls of microchannels and can be released by trypsin, resulting in label-free viable cells. This device has been suggested to be simple and low-cost using microreplication technologies and can also be automated [54]. This device is particularly suitable for high-throughput processing by using several high-aspect-ratio microchannels configured in parallel and can process 1 ml of input in less than half of an hour. A unique attribute of this microfluidic device is the ability to detect CTCs by using an integrated Pt conductivity sensor to detect the unique electrical properties of CTCs because the overexpression of surface proteins results in a higher conductivity than that of erythrocytes or leukocytes. Breast cancer cell line-spiked blood experiments (using Michigan Cancer Foundation-7 (MCF-7) cells) have been carried out, and the highest recovery was 97%; however, no studies have been conducted using the blood of patients with cancer as clinical samples [55]. The detection efficiency was approximately 100% for spiked samples. Due to the quantitative ability of the detector, no staining or cytometry is needed, allowing for approximately 100% recovery of mostly viable cells [54]; therefore, the obtained CTCs are suitable for molecular profiling using microchip technology designed for high recoveries [56]. HTMSUs can be used not only for separating CTCs but also for recognizing other molecules to target other rare cells [54]. To date, HTMSUs have been reportedly fabricated with EpCAM and prostate-specific membrane antigen (PSMA)-specific aptamers but can also be used for single-stranded nucleic acid oligomers, LNCaP-like cells [55], and bacteria (such as *E. coli* O157:H7) [54].

A CTC-Chip is a microfluidic device consisting of an array of 78,000 chemically functionalized (with the anti-EpCAM antibody) microposts within a 970-mm^2^ surface area [66]. Cell attachment to the antibody is promoted by the geometric arrangement of the microposts and fluid flow velocity. For optimal capture, a fluid flow rate of 1–2 ml/h is used for this device [30]. One of the disadvantages of microfluidic technologies is the low-throughput rate and therefore their inability to analyze large sample volumes. An approximately 60% recovery rate has been achieved with cancer cell line-spiked blood samples, and a similar result has been achieved with clinical samples from patients with cancer with approximately 98% cell viability [57]. A purity of 50% can be achieved with the CTC-Chip using the peripheral blood of patients with metastatic cancer (lung, prostate, breast, colon, and pancreatic cancers), and CTCs have been identified in 115 of 116 (99%) patient samples. Recent studies have demonstrated that EGFR mutational analysis can be carried out on DNA recovered from the chip [93].

A geometrically enhanced differential immunocapture (GEDI) chip is also a microfluidic device that uses geometrically enhanced differential immunocapture, which combines positive enrichment (using antibody-coated microposts) with hydrodynamic chromatography to minimize nonspecific leukocyte adhesion. The geometry of this device has been designed to maximize streamline distortion and thus bring CTCs in contact with immunocoated walls for capture [58]. With this solution, when cell-coated wall impact does not result in capture, the cells are displaced onto different streamlines depending on their size and collision inclination [30, 58]. This property of the GEDI chip can increase the purity of cell capture by decreasing unwanted interaction opportunities of nontarget blood cells with immunocoated surfaces. In the device, 5000 microposts have been fabricated in either a circular or octagonal shape (80 mm diameter) in a 100 × 8 × 25 mm channel [58]. The GEDI chip has been used to capture CTCs from anti-prostate-specific membrane antigen (PSMA) cell lines spiked into the blood, where the capture efficiency was ~ 85% with a purity of 68%. The GEDI chip has also been tested on castrate-resistant prostate cancer (CRPC) patients, where a 1 ml blood sample was processed. This method is also used to perform downstream analyses, such as cDNA sequencing and immunostaining, on cells isolated with the GEDI chip [30, 58].

#### Capture enhanced by nanomaterials

Interactions between CTCs and antibodies play an important role in cell capture. New techniques have enhanced the efficiency of immunoaffinity methods. It has been discovered that nanomaterials can enhance the capture efficiency and thus the similarities in size between a nanoparticle and cell membrane. Coating these nanoparticles with CTC-specific antibodies increases the surface area for CTC binding. Here, we describe approaches that utilize nanomaterials for enhanced CTC capture.

A graphene oxide (GO) chip is a microfluidic device that uses the unique properties of nanomaterials for more sensitive CTC capture. It utilizes functionalized graphene oxide nanosheets, which is a biocompatible nanomaterial with a high surface area that serves as a platform for sensitive CTC isolation, allows the imaging of captured CTCs, and enables culturing of the captured cells. The GO chip uses flower-shaped gold patterns (100 μm × 100 μm) as a base for the absorption of GO nanosheets. GO sheets are chemically functionalized with EpCAM antibodies and result in a high surface-to-volume ratio for capturing CTCs in a simple chamber-like structure without the need for three-dimensional structures, which make culturing and functional characterization difficult. The device dimensions are 24.5 mm × 60 mm × 3 mm. For spiking experiments, human breast cancer cell lines (high EpCAM-expressing MCF-7 cells and non-EpCAM-expressing Hs-578Tcells) and a low EpCAM-expressing human prostate cancer cell line (PC-3) were used in a buffer. The capture yield for EpCAM-expressing cells was over 80% for both cases, whereas for the non-EpCAM-expressing cells, the capture yield only reached 10%. The recovery rates for EpCAM-expressing cells were over 65% for all spiked samples. This method can be used for the isolation of CTCs from blood samples of patients with pancreatic, breast, and lung cancer [87]. Although the GO chip can be used to successfully isolate CTCs, it shares the following common problem with many immunoaffinity-based methods: the difficulty of releasing viable cells from the capturing surface. To overcome this problem, further improvement of the GO chip has been carried out using a thermoresponsive polymer, which allows for gentle CTC release, maximizing cell viability [60]. Thermoresponsive polymers belong to the class of stimuli-responsive polymers; thus, temperature changes cause conformational changes in the polymer [61]. The bottom substrate of the tunable thermal-sensitive polymer-GO chip is coated with functionalized GO that is spread over a matrix of thermoresponsive polymers. A higher capture efficiency (84.93–95.21%) has been achieved for EpCAM-expressing cancer cells, while for EpCAM-negative cells (Hs578T cells), the efficiency remained relatively low. The cell numbers showed a release of 95.21% and 91.56% in buffer and blood experiments, respectively, and the viability of released cells was 91.68%, as indicated by a live-dead assay. For blood tests, patients with different types of cancer were tested and resulted in 100% (*n* = 20; patients with metastatic breast cancer (*n* = 7), early-stage lung cancer (*n* = 4), and metastatic pancreatic cancer (*n* = 9)) [87]; 80% (10 patients with metastatic breast cancer); and 67% (three patients with pancreatic cancer) [60] capture efficiencies. This device has the advantages of easy fabrication, high purity, and the possibility of releasing cells to conduct various downstream analyses, such as genotyping, single-cell profiling, and standard clinical cytopathological analysis.

Another microfluidic device, which uses a silica nanoparticle (SiNP) platform to enhance the efficiency, is an EpCAM-coated patterned silicone nanopillar (SiNP) that increases the surface area for molecular interactions connected to a polydimethylsiloxane (PDMS) chip with a serpentine chaotic mixing channel that increases the frequency of possible interactions between substrates and CTCs in a sample [62]. The PDMS chip has serpentine chaotic mixing channels that contain chevron-shaped micropatterns embedded at the top of the channels, generating vertical flow that facilitates contact between CTCs and the substrate. Using EpCAM-positive cell cultures (MCF7, PC3, and T24 cells) in phosphate-buffered saline (PBS) or Dulbecco’s-modified Eagle’s medium (DMEM) at flow rates of 0.5, 1, and 2 mL/h, an efficiency of > 95% has been measured. The effect of capture rates has been investigated without the chevron-shaped micropatterns in the channels and SiNPs on the patterned substrate. In both cases, significantly lower cell capture was observed. A comparison study was conducted between the CellSearch system and the SiNP method by testing clinical blood samples (1 ml) of 26 patients with prostate cancer. In five cases, neither of the devices captured any CTCs, and in 17 out of 26 samples, the microfluidic SiNP platform captured significantly more CTCs than the CellSearch system [62].

The recently developed NP-^HB^CTC-Chip allows the release of cells with a chemical ligand-exchange reaction (gold nanoparticle (AuNP)-thiol exchange reaction) and has been engineered to release captured cells from the AuNPs, enabling subsequent CTC molecular analysis, such as next-generation RNA sequencing and ex vivo cell culture [63, 94]. Compared to the ^HB^CTC-Chip, the NP-^HB^CTC-Chip has shown an increased capture efficiency (ranging between 96.4 ± 2.2 and 80.0 ± 1%) and a decrease in nonspecific binding [94]. The surface can be coated with EpCAM or different surface markers, such as Her2 or EGFR, or a cocktail of the three surface markers, to achieve > 90% capture efficiency for low EpCAM-expressing cells (such as MDA-MB-231 cells). The efficiency remained high when low numbers of cells from different cell lines were analyzed. In clinical samples from four patients with metastatic breast cancer, CTC concentrations ranging from 6 to 12 CTCs/mL and a CTC cluster were discovered. The total processing time for 3 ml of whole blood is ~ 4 h. Cells released by glutathione (GSH) for thiol exchange remain highly viable and could undergo next-generation RNA sequencing. Unique breast cancer gene signatures have been found to provide useful clinical information [63].

#### Negative enrichment

Negative enrichment methods use an indirect approach by targeting antigens (e.g., CD45 and CD66b) that are on the surface of all blood cells except for CTCs. This method typically results in lower purity; however, the obtained CTCs are label-free and unbound, possibly containing all subpopulations because their antigens were not targeted [70, 79–81].

A negative enrichment-based strategy using immunomagnetic technology developed by StemCell™ is called EasySep™, where an enrichment cocktail containing the CD45 antibody and magnetic beads with various sizes are used for separation [64]. This kit has been tested with different beads on whole blood samples spiked with cancer cell lines. For all magnet separation conditions and samples, the mean log depletion of CD45+ cells was 2.9 ± 0.4, the mean recovery of cancer cells was 42 ± 23%, and residual red blood cell (RBC) contamination was ∼ 9000 RBC/ml. This kit is an easy-to-use and quick (25 min) negative immunomagnetic enrichment technique resulting in label-free viable cells, which can be used for subsequent downstream analyses [95].

The advantage of immunoaffinity techniques is high specificity. Only cancer cells containing distinct antigens, which bind to the antibody used for capture, are enriched. Considering tumor heterogeneity, this high specificity may be a disadvantage since only one subpopulation is captured; thus, information regarding other subpopulations of cancer cells is not available. Using multiple antibodies might overcome this difficulty. Another problem is the release of captured CTCs from the surface of a device. Several approaches are discussed above; however, the data that is available for comparisons of these approaches is scarce. Currently, there is no optimal technique, but new approaches might emerge soon.

### Size based

Physical methods, such as size-based enrichment methods, are independent of antigen expression on the surface of a cell. Therefore, these methods avoid the inaccuracy of heterogeneous antigen expression observed in CTCs [96]. These methods use physical and mechanical differences between CTCs and other cells found in blood samples. Among physical methods, size-based methods isolate CTCs depending on their increased size (9–19 μm). Sized-based techniques mainly use membrane microfilters, but other microfluidic-based techniques have also been reported [30, 72]. The main advantage of size-based techniques is that they result in label-free, unmodified and viable cells using a fast and simple method with a high capture efficiency and good enrichment (10^4^ against leukocytes) [32, 64–67, 70, 72, 95–97], which typically allows the obtained cells to be used for subsequent downstream methods, such as next-generation sequencing (NGS), to gain more information from one sample and improve the detailed analysis of a specific cancer type and cancer progression from patient to patient. Size-based techniques can have a much shorter enrichment time without biochemical modifications and are also expected to cost less because of the lack of expensive labels [98]. However, one of the largest challenges of size-based CTC isolation is possible interference with leukocytes (7–9 μm) [99], which have a size comparable to the lower range of CTC size (9–19 μm) [100]. Even smaller CTCs have been reported in several cancer cases, indicating that smaller CTCs are likely underrepresented in an enriched sample when using size-based methods [47]. Another challenge during validation is due to a size difference between CTCs and cancer cell lines, which are regularly used for preliminary validation. Compared to clinical samples from patients with cancer, using cells from cancer cell lines, which are significantly larger than CTCs, for validation results in irrelevant results. During the evaluation of filtration techniques, cell lines with a median size of 11–13 μm should be used to overcome this problem [99].

#### CTC membrane microfilters

Microfiltration methods have become available because of microfabrication methods that are used to construct thin, typically polycarbonate films containing controlled nano- to micron-sized pores (where pores are formed by surface bombardment) [101]. While track-etched filters are cost-effective and can be mass produced, unevenly distributed, low density or fused pores can reduce the CTC capture efficiency to 50–60% [101]. In the case of microfilters, purity is important not only because it allows detailed gene analysis but also because leukocytes can clog the filter, and when fluorescent imaging is used, it can increase the fluorescent background noise, which can cause false-positive results.

**Fig. 3:**
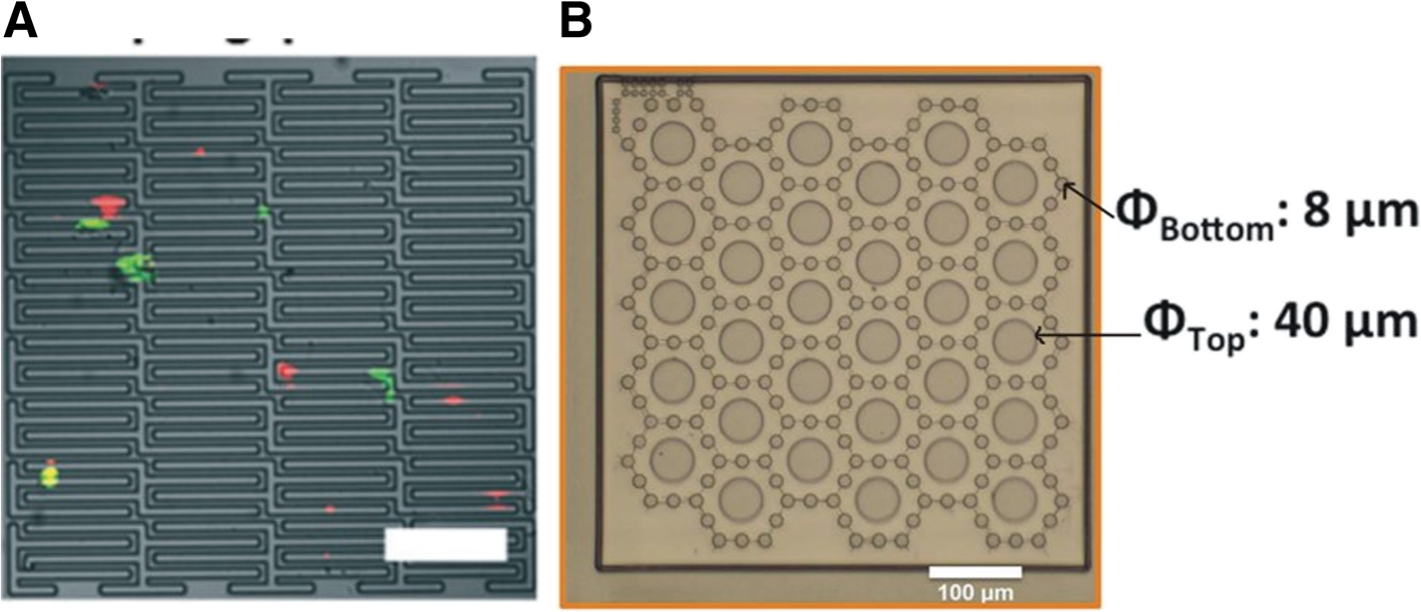
Different CTC membrane microfilters where **a** an FMSA contains highly porous and flexible micro spring structures in a single layer [32] and **b** shows a separable bilayer structure where capture is achieved by a gap between the top and bottom porous membranes with 8-μm-diameter holes arranged hexagonally on the bottom layer and larger holes with a diameter of 40 μm on the top layer [33]

A flexible micro spring array (FMSA) is a high-throughput device with a rapid processing speed for the separation of CTCs based on size and deformity [32, 67]. This device contains highly porous and flexible micro spring structures that are etched into a single layer of a parylene diaphragm, with an effective area of 0.5 cm^2^, as shown in Fig. 3a. This device is suitable for processing larger volumes of blood without clogging within 10 min. For round-shaped pores, a pore diameter of 8 μm has been found to have the best performance in this device. The FMSA also contains a pressure regulation system to prevent mechanical damage to cells during filtration. In spiked blood samples (using MCF-7, MDA-MB 231, C8161, and WM35 cell lines), the capture efficiency is 90% with greater than 10^4^ enrichment and greater than 80% viability. In clinical samples, the FMSA has been shown to detect CTCs in 16 out of 21 samples (76%). CTC microclusters can also be observed using this device. The captured cells have also been tested for vimentin, which is a marker for mesenchymal cells, and a substantial number of the cells (~ 86%) were positive, showing the advantage of size-based separation compared with immunoaffinity-based separation. CTCs caught on the filter maintain their viability and can be cultivated in the device or extracted by reverse flow. Genetic analysis, which can provide diagnostic and prognostic information can also be carried out on live cells [67]. Recently, a tandem flexible micro spring array (tFMSA) has been developed to specifically separate cells based on differences in sizes and deformability. The tFMSA was designed by integrating individual FMSA membranes in tandem and placing decreasing gap widths for the separation of up to four distinct subpopulations of viable cells [32, 65].

In a recent microfluidic device, multiple arrays of crescent-shaped isolation traps (or wells) have been used with two 5-μm gaps between them where the wells are alternated left and right relative to the flow stream to prevent clogging and allow each well to hold a single CTC or more CTCs with smaller sizes while allowing other blood constituents to pass through the device [68]. During the retrieval of cells, positive pressure is applied to reverse the flow direction. The round-shaped bases of the wells can decrease in size to minimize obstructions during backflow and decrease mechanical insults to cells colliding with the device. Pressure regulators control the flow rate and can make real-time changes to the flow characteristics in the device. Three different cell lines, including human adenocarcinomas (MCF-7 and MDA-MB-231 cells), human colon adenocarcinoma (HT-29 cells), gastric adenocarcinomas (AGS and N87 cells), hepatocellular adenocarcinomas (HepG2 and HuH7 cells), tongue squamous carcinoma (CAL27 cells), and pharynx squamous carcinoma (FADU cells), have been used for the testing of the device. The results showed at least 80% efficiency and 80% purity for every sample at a 5-kPa pressure at a 0.7-ml/h flow rate. Compared to normal cultured cells, the recovery of cells was greater than 90% for every cell type with no effect on the proliferating capacity. To address the clinical need for larger volumes of blood, up to 5 ml of a sample can be processed in three devices at once at a pressure of 5 kPa in 2.5 h [69]. This device can be used with a broad range of cancer types without any functional modifications and has been tested on several cell lines from different origins. This method has been shown to be reproducible when CTC counts from patients with metastatic lung cancer (*n* = 5) were measured using different amounts of blood from the same samples (1, 2, and 3 ml of whole blood) [102].

A separable bilayer (SB) microfilter has been recently developed for viable size-based CTC capture [33]. The SB microfilter has a fundamentally different device structure and filtration principles than typical microfilter devices. In the SB microfilter, capture is achieved by a gap between the top and bottom porous membranes. The membranes consist of the biocompatible polymer parylene-C, which better preserves cell viability. The bottom layer contains 8-μm-diameter holes that are arranged hexagonally, and larger holes with a diameter of 40 μm compose the top parylene-C layer and are aligned to the centers of the corresponding hexagonal patterns on the bottom layer, as shown in Fig. 3b. Unlike other single-layer CTC microfilters, the precise gap between the two layers and the pore alignment is designed to result in a drastic reduction in mechanical stress on CTCs to capture viable cells. An investigation of the operation of the SB microfilter showed that the majority of captured tumor cells are located along the edges of the large top pores; however, tumor cells could partially or completely wedge into the gap between the top and bottom parylene-C layers. After tumor cells are captured on the SB microfilters, it is possible to separate the top and bottom parylene-C membranes to access the captured cells. Using multiple cancer cell lines spiked in healthy donor blood for analysis, the SB microfilter showed a capture efficiency of 78–83%, cell viability of 71–74%, and tumor cell enrichment of 2–3 × 10^3^. This device has also been investigated in a metastatic mouse model, where SB microfilters successfully enriched viable mouse CTCs from 0.4–0.6 ml of whole mouse blood samples and established in vitro cultures for subsequent genetic and functional analyses [33]. The SB microfilter was further tested with 1 ml of clinical blood samples from patients with metastatic colorectal cancer. Notably, while the current SB microfilter device can process a whole tube of fresh blood (7.5 ml), the processing time is much longer, the blood has a higher risk of clogging the device, and the captured cells are too crowded to maintain a cancer cell morphology, which might prevent further analysis [33].

A lab-on-a-disc platform using fluid-assisted separation technology (FAST), which allows rapid size-based isolation of CTCs with relatively high purity from whole blood, has been recently developed and commercialized by Clinomics [34]. This device consists of a track-etched polycarbonate membrane with 8-μm pores that allows size-selective CTC isolation and membrane pores filled with a stably held liquid during the entire filtration process to reduce clogging and the separation time. During the entire filtration process with the FAST disc, the chamber under the membrane is filled with an aqueous phase; therefore, when the blood phase reaches the membrane, the blood phase flows down to the lower chamber uniformly and diffuses in the aqueous phase. The FAST disc uses the entire membrane area for filtration and greatly alleviates clogging issues. CTCs from whole blood can be isolated on the filter by spinning the disc, and the total filtration time required to isolate CTCs from 3 ml of whole blood is less than 1 min. For CTC counting, immunostaining can be conducted on the disc. This device has been tested on blood samples spiked with various cancer cell types, such as MCF-7, MDA-MB-231, and MDA-MB-436 breast cancer cell lines; HCC78 lung cancer cell lines; and the AGS gastric cell line. The capture efficiency was highest (> 95.9 ± 3.1%) at a spinning rate of 600 rpm, while the purity was 2.5-log depletion (mean 6420 white blood cells (WBCs)/ml; range 5748–7176 WBCs/mL) at the same spinning rate, and the recovery rate was 96.2 ± 2.6%. For clinical tests, 3 ml of whole blood was collected from patients with cancer and was used without dilution or RBC lysis. Using this method, the typical problem of blood samples from patients with cancer causing clogging is negligible when used in FAST mode operation. With the use of FAST disc, CTCs were detected in 83.3% (15 of 18) of breast cancer cases and 82.9% (63 of 76) of stomach cancer cases. Notably, all the patients with breast and stomach cancer in this study did not have distant metastases, while most of the studies discussed in this review used blood samples from patients with known metastatic cancer. These results clearly foreshadow the use of the device not only for cancer treatment but also for early diagnosis. A dramatic increase in the recovery rate can be achieved (from 54.0 ± 21.0% to 95.9 ± 3.1%) using the FAST disc compared to using a similar disk without FAST mode [34]. The FAST disc approach enables a standalone, efficient, user-friendly, robust, and cost-effective lab-on-a-disc system for point-of-care isolation of CTCs for CTC capture and further downstream molecular analysis, such as immunostaining, high-resolution imaging, and mutation analysis, which are particularly important for personalized therapy in cancer treatment processes [34].

#### Microfluidic sorting devices

Microfluidic devices typically contain antibodies that target cell surface antigens; however, this method results in a labeled and selected subpopulation of CTCs. To overcome difficulties caused by labeling and take advantage of microfluidic methods, a new technique has been introduced in which a microfluidic method is combined with microcavity arrays [72, 73].

A common problem with mechanical microfilters is clogging and adhesion of the blood sample. Increased fluid-driving pressure caused by the accumulation of cells on the filter increases the chance of damaging captured cells. Additionally, prolonged contact with the filter can cause irreversible adhesion of captured cells and decrease the number of obtainable cells, thus resulting in reduced efficiency and recovery [70]. Since this problem is associated with all methods using the microfluidic technique, several solutions have been used in different devices, which we will discuss when describing the techniques. In general, microfluidic design goals involve creating a single-step and cost-effective device preferably without the need for prelabeling or preprocessing of the sample.

**Fig. 4:**
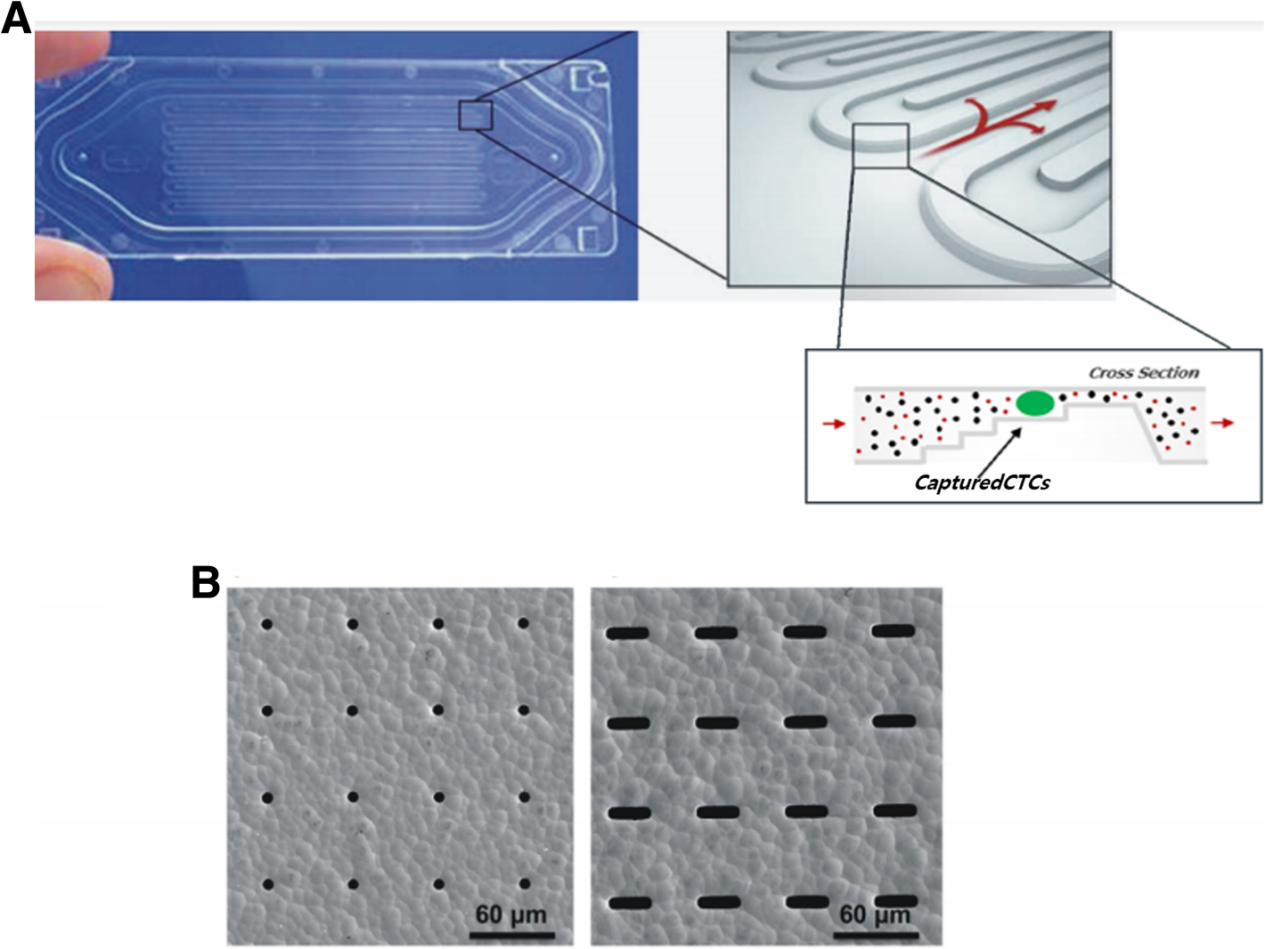
Microfluidic CTC sorting devices where **a** Parsortix contains a microscale stepped separation structure (image on the left side) with a cross-sectional gap that gradually decreases the dimension of the fluid path (schematic on the right side) [70, 107] and another microfluidic device and **b** an MCA contains microcavity arrays with circular- (image on the left side) or rectangular-shaped cavities (image on the right side) [31]

ANGLE developed a disposable microfluidic cassette called Parsortix that allows the collection and harvest of CTCs with a reverse flow [70, 71]. This device has a microscale stepped separation structure with a cross-sectional gap that gradually decreases the dimension of the fluid path and retains CTCs based on their less deformable nature and size, while letting other blood particles pass through the device, as shown in Fig. 4a. It can be fabricated with different arrangements (gap sizes ranging from 10 μm to 4.5 μm) to fit individual objectives and prevent clogging [70, 99, 103–105]. The main advantages of this approach are the delivery of both high purity and viable CTCs and plasma for cfDNA analysis, not only enabling further analysis of CTCs but also allowing for direct comparisons of molecular readouts from both cell DNA and cfDNA [106]. For the preliminary validation of the device, 4 ml of spiked blood samples using different cancer cell lines with different numbers of cells were investigated, and the capture rates were between the range of 42–70% for individual cell lines. In the same experiment, the harvest efficiency was also investigated, and the harvest efficiency of the individual cell lines was between the range of 54 and 69% [106]. After recovery of the cells, this device can provide viable CTCs in liquid suspension for subsequent molecular and functional characterization [70]. It has also been reported that the capture of CTC clusters is also possible with this device [107].

A microcavity array (MCA) [31, 72, 73] is another microfluidic system for the size-based separation of tumor cells that focuses on highly efficient and reproducible cell recovery. The MCA system is composed of a blood reservoir, filter-included cartridge, and individual tubes and uses a filtration method with metal filters consisting of nickel and gold via electroformation. In this device, the filtration cartridge is composed of polymethyl methacrylate (PMMA) and contains a metal filter that has a controlled pore shape, size, and density, overcoming the common problems of track-etched polycarbonate filters. The size of the microcavities has been optimized to trap tumor cells in the microcavities while allowing blood cells to flow through the device [72]. Two types of cavities have been developed for catching small-sized CTCs, such as small cell lung cancer (SCLC) cells (12.5-μm diameter), in circular (8-μm cavities [108]) or rectangular cavities (with either 5–9 μm × 30-μm cavities or 8 μm × 100-μm cavities [109]), as shown in Fig. 4b. The rectangular cavities were found to have a smaller pressure drop and captured significantly fewer leukocytes when the width of the microcavity was greater than 7 μm [108]. In earlier studies, the MCA device with circular 8-μm pores proved to be very efficient in capturing non-small cell lung cancer (NSCLC) cells using cell lines (~ 96% detection efficiency in 1-ml blood sample spiked with 10–100 cells), and it was observed to be superior to the standard CellSearch method in detecting CTCs in clinical studies from the blood of patients with NSCLC. The MCA system was also able to isolate CTC clusters that could not be completed by immunomagnetic separation systems [110].

The automated version of the previous device, which has a pumping system allowing for subsequent high-throughput analysis and decreased handling and is therefore more suitable for clinical use, has been recently developed [109] with an efficiency and reproducibility equal to or even superior to the abovementioned manual method [31, 72, 73]. The MCA has rectangular 8 × 100-μm pores inside a PMMA cartridge, which has been adapted for mass production. The automated system combines enrichment, staining, and washing steps in a single integrated microfluidic device that can simultaneously accommodate reagents and four independent samples. According to previously reported data, the MCA is suitable for the recovery of CTCs as well as for subsequent molecular analysis of blood obtained from patients with cancer [109]. In another automation development, a size-selective MCA has been integrated with a microfluidic device so that the enrichment of CTCs from blood as well as the staining and washing processes in the microfluidic assay can be performed within one integrated device [72]. Interestingly, a fast and simple method for entrapping and encapsulating a single CTC has been developed, where CTCs visible to the naked eye can be handled easily with tweezers without the contamination of neighboring cells. This method can be used for genetic mutation analyses by whole genome amplification (WGA) [108].

### Density based

Centrifugation, which uses the specific density of RBCs, leukocytes, and cancer cells, is one of the first reported methods for CTC isolation. To exploit these differences to separate these cell types, a silicone flotation technique has been used [93]. Currently, the use of buoyancy to separate different particles based on their relative densities is called density-based gradient centrifugation or isopycnic density gradient centrifugation [30].

Specifically, the OncoQuick**®** (GrenierBioOne, Frickenhausen, Germany) technique is designed for CTC isolation and has combined density-based gradient centrifugation and filtration by integrating a porous barrier into the system above the separation media, which captures CTCs while allowing erythrocytes and some leukocytes to pass through the device [74]. A study of OncoQuick revealed that following centrifugation, OncoQuick resulted in recovery rates of 87%. The increased depletion of mononuclear cells when using OncoQuick compared to another centrifugation technique has resulted in a simplified workflow for sample processing and immunocytochemical detection [74]. Another study revealed that OncoQuick can detect CTCs in 23% (14/61) of patients with metastatic cancer [111]. Several clinical studies have used OncoQuick for CTC enrichment [112, 113].

The AccuCyte system is fundamentally based on the density of CTCs, which is within the range of the buffy coat. However, it is different from existing density-based methods that separate the buffy coat from RBCs and plasma because a unique separation tube containing a lozenge-shaped float and collector device is used, allowing virtually complete harvesting of the buffy coat into a small volume for application to a microscopic slide without cell lysis or wash steps, which is a potential source of CTC loss [75]. The float is a hollow plastic cylinder with longitudinal ribs that are raised 75 microns on the surface to prevent contact of the float body with the inside wall of the tube, thereby providing channels for fluid movement during centrifugation. Centrifugation separates blood within a tube into a bottom layer of packed RBCs (the hematocrit), a top layer of plasma, and a buffy coat layer of white blood cells and platelets. After density separation, a CyteFinder system (an automated scanning digital microscope and image analysis system) can be used for the classification of CTCs. The test results of cancer cell lines showed an average recovery rate of 90%, while 22 out of 27 (81%) CTCs were detected in the samples. Clinical tests of patients with advanced breast, prostate, and colorectal cancer have also been carried out. After CTC enrichment, genomic analyses can be performed on individual CTCs using this method [75].

One of the main advantages of physical methods, such as size-based techniques, is that they result in label-free, unmodified and viable cells with a simple method, typically resulting in cells that can be used for subsequent downstream methods. Physical separation techniques can also result in a much shorter enrichment time and are expected to cost less without biochemical modifications. However, one of the largest challenges of size-based CTC isolation techniques is the possible interference with other cells, such as leukocytes, that have a size comparable to the lower range of CTC size, while another challenge can occur during validation because of the size difference between CTCs and cancer cell lines, which are regularly used models.

### Combined

A CTC-iChip consists of two separate chips (a CTC-iChip1 and a CTC-iChip2) that are organized for inline operation combining three technologies [100]. The first chip uses continuous deterministic lateral displacement (DLD) with an array of droplet-shaped posts with 20 or 32-μm gaps for a hydrodynamic size-based separation of nucleated cells (WBCs and CTCs) from whole blood. Then, the nucleated cells flow through a microchannel and are aligned by inertial focusing. The next phase is the immunomagnetic isolation of CTCs with microfluidic magnetophoresis. Cells tagged with magnetic beads are driven into a collection channel. The CTC-iChip is capable of isolating cells in a tumor antigen-dependent (positive selection) or tumor antigen-independent (negative selection by depletion of WBCs tagged with magnetic bead-conjugated CD45, CD16, and CD66b antibodies) mode; therefore, it is suitable for sorting CTCs of various cancers. The tumor antigen-independent mode provides benefits of yielding viable label-free high-purity cells with both epithelial and mesenchymal characteristics for subsequent characterization (such as transcriptome analysis via NGS and high-quality clinically standardized morphological and immunohistochemical analyses) [35, 114].

While this technology overcomes the problems of simple size-based and immunomagnetic separation technologies, the shortcomings of this system are its long setup times and separate manually interconnected chips that are difficult to use in a clinical environment. Fachin et al. [76] developed an automated monolithic CTC-iChip that combines the three technologies on a single mass-producible plastic to improve accessibility by reducing the operating time and technical requirements. In this device, CTCs spend less than 8 s in the chip, while the depletion of blood cells occurs at 15–20 million cells per second. To show the advantages of both antigen- and size-independent selection methods, patient-derived CTCs (breast, prostate, and lung cancers and melanoma) selected by the monolithic CTC-iChip have been analyzed for surface marker expression and size. These results also confirmed previous reports that CTCs from patients with cancer and CTCs from cancer cell lines (used to spike blood for testing) vary in size and that EpCAM expression depends on individual cells [76].

Another combined method has been introduced [77], where a negative immunomagnetic method is combined with a microfluidic negative selection platform. This technique provides centrifugation-free WBC depletion and a chemical-free RBC depletion approach without manual sample transfer, making it suitable for possible future clinical use. For separation, in the first step, immunomagnetic WBC depletion is employed directly from 2 ml of whole blood by adding the CD45 antibody and 1-μm magnetic beads. The separation in this step is carried out by external magnets that are placed around a syringe barrel containing the sample mixture. Then, in the second step, WBC-depleted blood flows through a microfluidic chip from a second syringe barrel that contains a precision-manufactured micro slit membrane that is designed to selectively allow RBCs and platelets to pass through the device while retaining nucleated cells. Additionally, the microfluidic chip can also be used for inspection by microscopy for detection. Greater than 90% WBC depletion and greater than 90% recovery of CTCs within 1 h can be achieved by this simple and easy-to-use assay. This system has been investigated with both cancer cell line-spiked samples and with samples from patients with cancer, where CTCs were successfully detected in all 15 patient blood samples. Since blood samples are not subjected to any chemical manipulation during the separation process, CTCs can be subsequently used for molecular profiling [77].

What can we conclude from Tables 1–2? First, the deviation of the parameters is wide. The purity is approximately between 42–100%. The sample volume is between 0.5–9 ml. The clinical detection rate is between 23–100%. Interestingly, considering the methods where the clinical detection rate is available, if the capture efficiency is greater than 60%, the clinical detection rate could achieve 100% regardless of the purity. For clinical application, the most important parameters are the sample volume and the clinical detection rate. Considering a similar detection rate, lower sample volumes result in better clinical tests since CTC sampling can be repeated. Currently, several methods have the same detection rate; however, not all the methods were tested in clinical trials. Finally, the actual value of the CTC techniques for patient care remains not thoroughly researched, but the expectation is high among clinicians in the field of individualized therapy and/or therapeutic and prognostic markers. Considering the intensive methodological research of CTCs, it is currently not known which technique, if any, will become the standard.

## Future directions: downstream analysis

Liquid biopsy is a minimally invasive technique that can be repeated nearly limitlessly, making the real-time monitoring of tumor progression a possibility by using CTC counting techniques [115]. Although CTC counting is a promising method for early cancer detection, development, progression, and treatment, combined with molecular profiling, it can lead to the detection of CTC subpopulations, cancer subtypes, and gene mutations causing cancer development, progression, and drug resistance. Therefore, it can justify the increase in precision medication [116]. With the use of precise and high-throughput DNA analysis techniques, such as NGS, it is currently possible to describe the whole genome and transcriptome of a single CTC [117, 118]. The automation of NGS analysis of a single CTC also makes it possible to analyze whole CTC populations [114, 119, 120]. cfDNA and NGS are two potential tools for personalized medicine. cfDNA is mainly a prognostic and diagnostic biomarker, while NGS is used for mutational analysis of tumors. The total amount of cfDNA varies from patient to patient in the plasma or serum, but the average is higher in patients with cancer than in those without cancer. One of the main features of NGS is parallel sequencing, which is an advantage when several patient samples or genomic regions are processed or compared. Moreover, when multiple targets must be analyzed, NGS can reduce the cost. For cfDNA, plasma, serum, and other bodily fluids (e.g., urine, pleural fluid, and ascites) can be used; for NGS fine-needle aspirates, tumor excision or formalin-fixed paraffin-embedded (FFPE) tumor tissues can be used. Thus, it is typically easier to obtain samples for cfDNA than for NGS.

For further downstream analysis and molecular profiling, not all CTC isolation techniques can be used to improve the clinical outcome of patients with cancer. To combine CTC counting with DNA analysis, the CTC method that is used must result in viable cells after retrieval [30]. Since we have found it important to highlight the suggested future clinical use of CTC isolation and analysis methods, we would also mention CTC enrichment methods that use whole blood samples and are suitable for subsequent downstream analysis.

## Conclusions

Various methodological approaches of new early diagnostic techniques for patients with cancer were reviewed. Various methods for the separation and detection of CTCs from whole blood by CTC technologies were described. As described, these CTC strategies have a great advantage in terms of noninvasive or minimally invasive diagnosis by liquid biopsy that improves the comfort of patients with cancer by avoiding unnecessary and invasive sampling of a tumor. Although clinical utilization remains rare for CTC techniques, only one method has been approved by the FDA to date. Various approaches for clinical use in cancer diagnosis and metastasis monitoring are available, especially since animal studies have shown improved survival rates in animal studies when CTCs were removed from circulating blood [121].

This review presented the latest technologies that enhance the clinical significance of CTC separation techniques and presented various methods, including immunoaffinity, size-based, and combined methods. Technologies have been introduced with positive and negative enrichment methods, some of which remain in the verification phase, but others have already been licensed [34, 50, 51, 53, 64, 70, 71, 74, 75]. Because of the variety of CTC separation technologies and products that are currently in development, it is difficult to evaluate and compare all CTC techniques to each other and propose one or few of these techniques as standard approaches in oncology. In addition, it is difficult to compare CTC techniques since they have been applied for different cancers and use different separation methods, and the results have been interpreted differently. However, several methods mentioned in this review can not only be used for CTC isolation but can also be adjusted for capturing different cells from samples, which can make these techniques relevant for application in other fields focusing on cell separation.

For future use and general acceptance by physicians, patients, and governments responsible for healthcare, it is necessary to obtain reliable clinical data for the standardization and reproducibility of CTC technologies, which is already in progress for most of the described methods. Based on our comparison of various methodological approaches, we suggest some directions for the development of future technologies for clinical needs.

The capture yields of positive immunoaffinity methods are only high for specific antigen-expressing cells that are used for capture and are low for other cancer cell types. Thus, immunoaffinity methods provide sufficient information regarding cancer cell heterogeneity. Therefore, immunoaffinity methods using several antigens concomitantly should be further developed. One of the advantages of negative enrichment is that the obtained cancer cells are label-free. Thus, this approach provides information on the heterogeneity of CTCs. In fact, there are no data available that compare the heterogeneity of CTCs and primary tumors. It is possible that CTCs, which represent metastatic cells, are more homogenous than primary tumors due to clonal selection. Since biological therapy aims to target metastatic cells, liquid biopsy data are more appropriate for therapeutic decision-making than samples of primary tumors. Moreover, it is sometimes technically impossible to obtain fine-needle aspiration (FNA) samples from metastatic lesions, but a liquid biopsy to obtain metastatic cells is always available.

For clinical application, the length of time, ranging from 10 min to 2.5 h per sample, that is needed to produce test results is an important factor since the range of these applications is quite broad. Automation can be a key step in achieving higher throughput.

According to the available data, it is likely that CTC techniques using whole blood will become a standard procedure. Minimal or no sample preparation is required; therefore, tumor cell damage is minimized, resulting in more reliable CTC analysis. The usefulness of this technique is dependent on clinical data, where a liquid biopsy obtained as a diagnostic tool or predictive marker for therapy and prognosis. Since the speed of technical advancement is quick, much less clinical data are available. However, experimental data predict breakthroughs of this technique regarding clinical practice.

## Abbreviations

AGS and N87: Gastric adenocarcinomas
CAL27: Tongue squamous carcinoma
cfDNA: Cell-free DNA
CRPC: Castrate-resistant prostate cancer
CTC: Circulating tumor cell
DBCO-Ab: Dibenzocyclooctyne group-modified antibody
EpCAM: Epithelial surface tumor marker
EMT: Epithelial-to-mesenchymal transition
EGFR: Epidermal growth factor receptor
FADU: Pharynx squamous carcinoma
FAST: Fluid-assisted separation technology
FMSA: Flexible micro spring array
HepG2, HuH7: Hepatocellular adenocarcinomas
HER2: Human epidermal growth factor receptor 2
HT-29: Human colon adenocarcinoma
HTMSU: High-throughput micro sampling unit
IMSs: Immuno-magnetosomes
LMNCs: Leukocyte membrane fragments resulting in a magnetosome
MCA: Microcavity array
MCF-7: Michigan Cancer Foundation-7
MDA-MB-231: Human adenocarcinoma
MUC1: Mucin 1
MACS: Magnetic cell separation system
MNCs: Magnetic nanoclusters
NGS: Next-generation sequencing
PIK3CA: Phosphatidylinositol 3-kinase catalytic
PDMS: Polydimethylsiloxane
PMMA: Poly methyl methacrylate
PSMA: Prostate-specific membrane antigen
RBC: Red blood cell
SiNP: Silica nanoparticles
SB: Separable bilayer
tFMSA: Tandem flexible micro spring array
